# Characterizing neutral and adaptive genomic differentiation in a changing climate: The most northerly freshwater fish as a model

**DOI:** 10.1002/ece3.4891

**Published:** 2019-01-15

**Authors:** Kathleen G. O'Malley, Felix Vaux, Andrew N. Black

**Affiliations:** ^1^ Coastal Oregon Marine Experiment Station, Hatfield Marine Science Center, Department of Fisheries and Wildlife Oregon State University Newport Oregon; ^2^ Present address: Center for Genome Research and Biocomputing Oregon State University Corvallis Oregon

**Keywords:** Arctic charr, climate change, genotyping‐by‐sequencing, population genomics, salmonids, Svalbard

## Abstract

Arctic freshwater ecosystems have been profoundly affected by climate change. Given that the Arctic charr (*Salvelinus alpinus*) is often the only fish species inhabiting these ecosystems, it represents a valuable model for studying the impacts of climate change on species life‐history diversity and adaptability. Using a genotyping‐by‐sequencing approach, we identified 5,976 neutral single nucleotide polymorphisms and found evidence for reduced gene flow between allopatric morphs from two high Arctic lakes, Linne'vatn (*Anadromous, Normal*, and *Dwarf*) and Ellasjøen (*Littoral *and *Pelagic*). Within each lake, the degree of genetic differentiation ranged from low (*Pelagic* vs. *Littoral*) to moderate (*Anadromous *and *Normal* vs. *Dwarf*). We identified 17 highly diagnostic, putatively adaptive SNPs that differentiated the allopatric morphs. Although we found no evidence for adaptive differences between morphs within Ellasjøen, we found evidence for moderate (*Anadromous *vs. *Normal*) to high genetic differentiation (*Anadromous *and *Normal* vs. *Dwarf*) among morphs within Linne'vatn based on two adaptive loci. As these freshwater ecosystems become more productive, the frequency of sympatric morphs in Ellasjøen will likely shift based on foraging opportunities, whereas the propensity to migrate may decrease in Linne'vatn, increasing the frequency of the *Normal* morph. The *Dwarf* charr was the most genetically distinct group. Identifying the biological basis for small body size should elucidate the potential for increased growth and subsequent interbreeding with sympatric morphs. Overall, neutral and adaptive genomic differentiation between allopatric and some sympatric morphs suggests that the response of Arctic charr to climate change will be variable across freshwater ecosystems.

## 1 INTRODUCTION

The Arctic has been profoundly affected by climate change within a brief period of time, with sea surface temperatures increasing significantly faster than the global average, and sea ice extent and thickness declining rapidly (Intergovernmental Panel on Climate Change, 2013). Further temperature increases will advance the break‐up of ice (Prowse et al., 2011), expanding the open period for some of the largest rivers in the world (Wrona et al., 2006). Accelerated glacial melting will also contribute to rising sea level and a higher frequency of landscape‐altering, glacial lake outburst floods (Harrison et al., 2018). Other stressors such as freshwater acidification, eutrophication, and ozone depletion will be exacerbated by climate change as well (Adrian et al., 2009; Heino, Virkkala, & Toivonen, 2009; Schindler & Smol, 2008; Woodward, Perkins, & Brown, 2010).

Prolonged climate change is expected to have a significant impact on Arctic freshwater ecosystems (Woodward et al., 2010), and have wide‐ranging effects on freshwater and anadromous Arctic fish species (Reist, Wrona, Prowse, Dempson et al., 2006; Reist et al., 2006a, 2006b; Rouse et al., 1997; Wrona et al., 2006). Elevated temperatures, increased light penetration, and a higher concentration of atmospheric carbon dioxide are likely to increase the productivity of lotic and lentic systems and cause shifts in habitat availability and quality across the region (Adrian et al., 2009; Brander, 2010; Heino et al., 2009; Michelutti, Wolfe, Vinebrooke, Rivard, & Briner, 2005; Prowse et al., 2006; Woodward et al., 2010). Novel competitors, prey, parasites, and diseases from lower latitudes are likely to invade with increased temperatures (Davidson et al., 2011; Fossheim et al., 2015; Parkinson & Butler, 2005). In turn, such a changeover in diversity is likely to modify foraging preferences and the trophic structure of Arctic fish (Reist et al., 2006a), which will affect commercial and subsistence fishing yields, as well as food safety (Davidson et al., 2011; Reist et al., 2006a; White, Gerlach, Loring, Tidwell, & Chambers, 2007).

The Arctic charr *Salvelinus alpinus* (Linnaeus, 1758) has a broad, circumpolar distribution and is the most northerly distributed freshwater fish species (primarily above 60°N; Klemetsen, 2010; Wilson et al., 2004). Consequently, Arctic charr may provide a useful model to study the impact of climate change on cold water adapted fish species and Arctic freshwater ecosystems in general. Although some models predict that small temperature increases (<2.5°C), may increase Arctic charr growth rates (Budy & Luecke, 2014; Elliott & Elliott, 2010; Reist et al., 2006a), Lehnherr et al.. (2018) have attributed a rapid decline in physiological condition of Arctic charr in Lake Hazen on Ellesmere Island to climate change. Furthermore, studies indicate that the eggs of the Arctic charr are not expected to survive a freshwater temperature increase of 5°C, and temperatures exceeding 22–23°C will likely result in mass adult mortality unless cold water refugia are present (Elliott & Elliott, 2010). Range expansion of competitive salmonid species into the Arctic is likely to cause a substantial decrease in the geographic range of the species (Ficke, Myrick, & Hansen, 2007; Reist et al., 2006a), with a 73% reduction predicted in Sweden by 2100 (Hein, Öhlund, & Englund, 2012).

At high Arctic latitudes, lakes are typically of recent postglacial origin with low biodiversity, and the Arctic charr is often the only freshwater fish species present (Christiansen & Reist, 2013; Power, Reist, & Dempson, 2008). Arctic charr exhibit complex migratory behavior that has likely aided their colonization of such remote regions, including the Svalbard Archipelago (Svenning, Klemetsen, & Olsen, 2007). For instance, some individuals complete their entire life cycle in freshwater (residency), whereas others hatch and grow as juveniles in freshwater, migrate to sea (anadromy) to forage for weeks or months, and then return to freshwater to overwinter or reproduce (Craig, 1989; Gilbert, Donad, Swanson, & Tierney, 2016; Jensen & Rikardsen, 2008; Moore, Harris, Tallman, & Taylor, 2013; Radtke et al., 1996; Tallman, Roux, & Martin, 2018). This life history is different from anadromy in Pacific salmon (*Oncorhynchus*) because all migrants, including juvenile fish, return annually to freshwater to overwinter. Notably, Arctic charr populations in the southern range of the species distribution do not exhibit anadromy (Klemetsen et al., 2003). This phenomenon has been attributed to increased productivity in lower latitude lakes, which negates the energetic benefit of migrating to feed at sea where the risk of predation is higher (Finstad & Hein, 2012; Jensen, Finstad, & Fiske, 2018).

Across the geographic range of Arctic charr, differences in migratory behavior, development, habitat usage, and feeding are frequently associated with seemingly distinct morphological variants in body size, shape, and coloration (Alekseyev, Samusenok, Matveev, & Pichugin, 2002; Berg, Finstad, Olsen, Vegar Arnekleiv, & Nilssen, 2010; Danzmann, Ferguson, Skùlason, Snorrason, & Noakes, 1991; Fraser, Adams, & Huntingford, 1998; Gíslason, Ferguson, Skúlason, & Snorrason, 1999; Hawley, Rosten, Christensen, & Lucas, 2016; Hooker et al., 2016; Knudsen, Klemetsen, Amundsen, & Hermansen, 2006; May‐McNally, Quinn, Woods, & Taylor, 2014; Parsons, Sheets, Skúlason, & Ferguson, 2011; Skùlason, Noakes, & Snorrason, 1989; Snorrason et al., 1994). These “morphs” can occur in sympatry or allopatry, and the number of morphs within a single freshwater system varies (e.g., Alekseyev et al., 2009; Gíslason et al., 1999; Hawley et al., 2016; Hooker et al., 2016; Knudsen et al., 2006; Parsons et al., 2011; Skùlason et al., 1989). For example, Lake Skogsfjordvatn in Norway contains three resident morphs: nearshore spawning individuals that eat a wide variety of prey (littoral omnivores), lake bottom spawning individuals that eat fish (profundal piscivores), and lake bottom spawning individuals that eat prey living on sediments (profundal benthivores; Skoglund, Siwersson, Amundsen, & Knudsen, 2015).

The degree of genetic differentiation among Arctic charr morphs is an ongoing topic of research, and their distinction has mostly been investigated using neutral microsatellites and mitochondrial genes. Past studies that did not consider sympatric morphs often found that individuals from different lakes were distinguishable from one another (Brunner, Douglas, & Bernatchez, 1998; Primmer et al., 1999; Wilson et al., 2004), particularly where lakes are landlocked and geographically isolated (Bernatchez, Rhydderch, & Kircheis, 2002; Shikano, Järvinen, Marjamäki, Kahilainen, & Merilä, 2015). However, low genetic differentiation reported among anadromous individuals from some locations hundreds of kilometers apart likely reflects recent postglaciation colonization events over long distances (Alekseyev et al., 2009; Moore et al., 2013; Wilson et al., 2004). Genetic admixture among nearby, connected lakes and rivers also indicates that some anadromous individuals migrate and reproduce outside of their source population (Bernatchez, Dempson, & Matin, 1998). A reduction in observed heterozygosity (the Wahlund effect) and a high allelic diversity within many lakes has implied population substructure that may correspond to sympatric morphs (Bernatchez et al., 1998, 2002; Primmer et al., 1999; Wilson et al., 2004). Studies focused on the distinction of sympatric morphs have become more common, and results from different lakes indicate that the degree of genetic differentiation varies from very low to complete (reviewed in Jonsson & Jonsson, 2001, e.g., Adams, Wilson, & Ferguson, 2008; Alekseyev et al., 2009; Alekseyev, Gordeeva, Matveev, Vokin, & Yur'ev AL., 2014; Danzmann et al., 1991; Gíslason et al., 1999; Gordeeva, Alekseyev, Matveev, & Samusenok, 2015; May‐McNally et al., 2014; Samusenok et al.., 2006; Wilson et al., 2004;). Overall, the genetic differentiation estimated among allopatric and sympatric morphs, as well between anadromous and resident individuals, appears to vary significantly across the entire range of Arctic charr.

It is currently uncertain how Arctic charr morphs will be affected by climate change. As freshwater productivity increases with climate change, resident morphs are predicted to increase in frequency, with fewer anadromous migrants occurring (Finstad & Hein, 2012; Reist, Wrona, Prowse, Dempson et al., 2006). This change may result in a reduced rate of gene flow and an increased degree of genetic differentiation among allopatric populations. Elevated freshwater temperatures and an increase in ice‐free days are also predicted to lengthen the potential growing season for Arctic charr (Elliott & Elliott, 2010; Reist et al., 2006a), which may cause small‐sized morphs to grow larger (Budy & Luecke, 2014). This size shift could increase the intensity of competition among small and larger sized sympatric morphs (Budy & Luecke, 2014) and result in more matings between morphs, eroding their genetic differentiation.

To investigate how Arctic charr may respond to climate change, we focus on morphs from two high latitude lakes, Linne'vatn and Ellasjøen, in the Svalbard archipelago, a Norwegian territory in the Arctic Ocean. An improved understanding of the genetic diversity within and among these morphs should indicate the adaptability of Arctic charr in Svalbard and other high latitude locations to factors such as increased freshwater temperatures and ice‐free days. Using a genotyping‐by‐sequencing (GBS) approach (Elshire et al., 2011), we identify both neutral and putatively adaptive loci and test for genetic differentiation among the sympatric and allopatric morphs to determine the degree of reproductive isolation that can be attributed to genetic drift or local adaptation.

Linne'vatn contains three morphs: *Anadromous*, typical‐sized *Normal* resident, and *Dwarf* resident fish. The morphs are all generalist feeders, although large individuals belonging to the *Anadromous *and *Normal *morphs are mainly cannibals, whereas small individuals (primarily the *Dwarf *morph) mostly eat insect larvae in open water (zooplanktivory; Svenning et al., 2007). Since climate change is predicted to increase freshwater productivity, potentially leading to a reduction or total loss of anadromy (Finstad & Hein, 2012; Jensen et al., 2018), we test for evidence of gene flow between the *Anadromous *and two resident morphs. If *Anadromous *individuals represent a distinct gene pool from resident fish, the propensity to migrate could be lost entirely among Linne'vatn Arctic charr. Increased temperatures may also lengthen the growing season of small‐sized Arctic charr (Budy & Luecke, 2014; Elliott & Elliott, 2010; Reist et al., 2006a), such that *Dwarf *individuals in Linne'vatn reach larger body sizes. This size change could increase the intensity of competition and rate of interbreeding between the *Dwarf *and typical‐sized *Anadromous *and *Normal *morphs, eroding any potential genetic differentiation and possibly leading to the extinction of the *Dwarf *morph.

In Lake Ellasjøen, a large‐sized, nearshore *Littoral* benthivore morph coexists with a small‐sized *Pelagic *zooplanktivorous morph (Hawley et al., 2016; Hawley, Rosten, Haugen, Christensen, & Lucas, 2017). A third dwarf morph (profundal benthivore) also occurs in Ellasjøen (Hawley et al., 2016), but was not included in this study. Our sample size was limited from Ellasjøen, but as a preliminary investigation, we tested for gene flow between the *Littoral *and *Pelagic *morphs. Climate change is likely to increase freshwater temperatures and productivity, facilitating the invasion of novel prey and parasite species (Davidson et al., 2011; Fossheim et al., 2015). This change in diversity is likely to modify the trophic structure of Lake Ellasjøen, altering foraging preferences and intensity of competition between the *Littoral *and *Pelagic *morphs. In turn, such change may affect the relative frequency of the two morphs. If gene flow is limited between *Littoral *and *Pelagic *fish, then each morph will need to adapt independently to the effects of climate change, or face extinction.

## 2 MATERIALS AND METHODS

### 2.1 Field sites and sample collections

**Figure 1:**
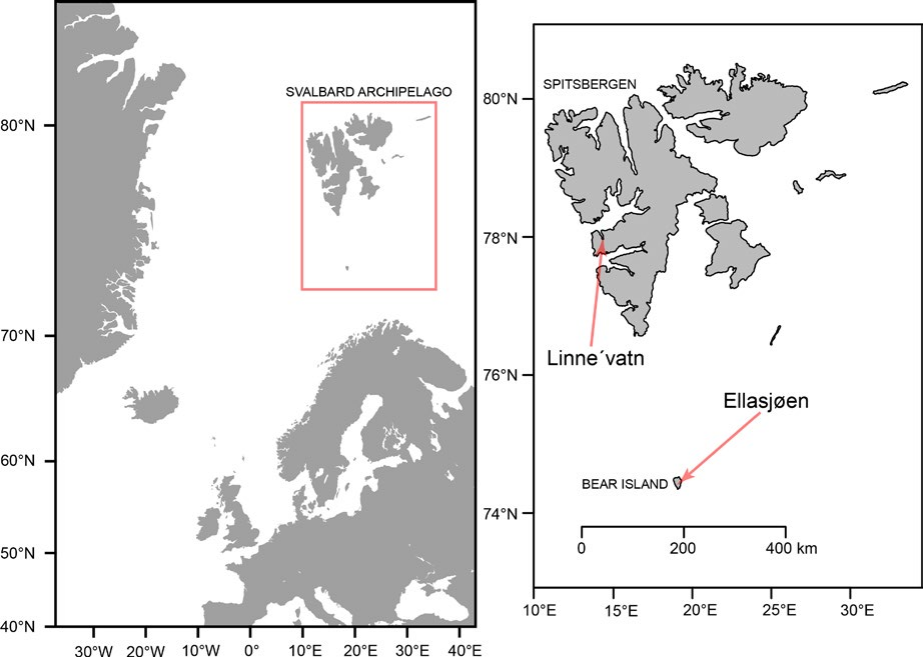
Location of the two lakes, Linne'vatn and Ellasjøen in the Svalbard archipelago. *Anadromous *Arctic charr were sampled in the Linne'vatn River, and both resident *Normal* and resident *Dwarf* charr were sampled from Lake Linne'vatn. *Littoral *and *Pelagic* Arctic charr samples were collected from Lake Ellasjøen. The inset map for the Svalbard archipelago was generated using the R package maps (Becker & Wilks, 2008)

Linne'vatn (78°03′N, 13°50′E) is located on western Spitsbergen, the largest island in the Svalbard archipelago (Figure 1). It is the second largest lake on Svalbard with an area of 4.6 km^2^ and a maximum depth of 37 m, occurring ~10 m above sea level (Bøyum & Kjensmo, 1978). There is a brief ice‐free period from late July to mid‐August, and the high water flow in the 2‐km long river between the lake and the coast allows access to the sea (Bøyum & Kjensmo, 1978). Lake Ellasjøen (74°30′N, 19°00′E) is located on Bear Island, a remote smaller island, equidistant between Spitsbergen and mainland Norway. The lake is 21 m above sea level with an area of 0.73 km^2^ and a maximum depth of 43 m (Bertram & Lack, 1933; Klemetsen, Grotnes, Holthe, & Kirstoffersen, 1985). Lake Ellasjøen flows into the short River Fossåa, but the lake outlet is too steep to permit the migration of Arctic charr (Klemetsen et al., 1985). The ice‐free period occurs from late June to mid‐September or October (Bertram & Lack, 1933).

Anadromous and resident fish were sampled from Linne'vatn in August 2013. Anadromous fish were captured in the main inlet river using a box trap net while resident fish were captured in the lake using a gill net. Two gill nets were used, the first of which had dimensions 1.5 m high and 30 m long with a mesh size of 26–39 mm, whereas the second net was 1.5 m high and 40 m long with a mesh size 10–45 mm. Both nets were used at a depth of 0–5 m in the littoral zone. Fork length (FL) measurements and sex (M/F) were recorded for each individual. A small piece of the adipose fin was removed from each fish and stored in a 1.5‐ml vial filled with 95% ethanol. In total, 24 *Anadromous* fish (FL range: 315–587 mm; M/F = 12/12) were collected from Linne'vatn River as they migrated from the sea to the lake. Resident fish were classified as either a *Dwarf *or *Normal *morph based upon FL, morphology, and sexual maturity. Fish caught in the lake were dissected to determine sexual maturity. Fish that were sexually mature and <205 mm were classified as *Dwarf* charr. According to these criteria, twenty *Normal *(FL range: 207–540 mm; M/F = 5/15) and 28 *Dwarf* (*N* = 28; FL range: 109–204 mm; M/F = 9/19) samples were collected from Lake Linne'vatn (Table 1).

**Table 1:** Arctic charr sampling information and summary statistics for filtered loci (8,075 SNPs) identified within each morph

Sampling information				Summary statistics			
Lake	Location	Morph	*n*	*H*_O_	*H*_E_	*F*_IS_	*A*_R_
Linne'vatn	River	*Anadromous*	22	0.2304	0.2354	0.0211*	1.585
	Lake	*Normal*	20	0.2254	0.2341	0.0372*	1.585
	Lake	*Dwarf*	28	0.2261	0.2358	0.0411*	1.594
Ellasjøen	Lake	*Littoral*	13	0.1499	0.1536	0.0242*	1.373
	Lake	*Pelagic*	6	0.1547	0.1593	0.0291*	1.384

* Heterozygote deficit (*p* < 0.001).

Arctic charr were sampled from Lake Ellasjøen in 2009 as part of a previous study (Hawley et al., 2016). In total, 19 resident individuals were obtained for genetic analysis. From these samples, two morphs were categorized based upon behavioral and physiological results from Hawley et al. (2016): 13 *Littoral* (FL range: 308–505 mm; M/F = 3/6) and 6 *Pelagic *samples (FL range: 212–314 mm; M/F = 2/1; Table 1). Sex was not recorded for seven Lake Ellasjøen samples.

### 2.2 Library preparation and sequencing

DNA extraction, library construction, and sequencing were conducted at Oregon State University's Center for Genome Research and Biocomputing, largely following the methodology of Elshire et al. (2011); modifications included the incorporation of a second restriction enzyme and a size selection step (see below). Briefly, genomic DNA was isolated from ~50 mg of tissue using a DNeasy Blood and Tissue Kit (Qiagen) and normalized to 20 ng/µl. Each sample was double digested in a 20 µl reaction using high fidelity *HindIII* and *MspI* restriction enzymes (New England Biolabs) at 37°C for 2 hr. Following digestion, 91 barcoded adapters and a common adapter were ligated to individual samples using T4 Ligase (New England Biolabs) at 22°C for 2 hr, and heat inactivated at 65°C for 30 min. All samples were pooled and cleaned using QIAquick PCR Purification Kit (Qiagen) and 300–500‐bp fragments isolated on a Blue Pippin (Sage Science). Size‐selected fragments were then amplified with an Illumina primer under the following conditions: 98°C 30″; 98°C 10″, 68°C 30″, 72°C 30″ (15 cycles); 72°C 5′, 4°C hold. The PCR product was purified, eluted, and quantified using a Qubit fluorometer (Thermo Fisher Scientific). Quality was assessed on an Agilent 2100 Bioanalyzer and loading concentration determined by qPCR. The final library was run on one Illumina HiSeq 3000 lane, using paired‐end 150‐bp sequence chemistry. Only single‐end reads were used for the current study.

### 2.3 SNP calling and quality filtering

Raw Illumina reads were first assessed for quality using fastqc (v. 0.11.5 Babraham Bioinformatics, Babraham Institute). The entire read length was retained as a visual examination of terminal read quality showed no substantial decrease in base call accuracy (median terminal base Q score = 32). Raw reads were then demultiplexed and constructed into putative genetic loci using the stacks pipeline (v.1.35; Catchen, Amores, Hohenlohe, Cresko, & Postlethwait, 2011; Catchen, Hohenlohe, Bassham, Amores, & Cresko, 2013). The *process_radtags* script in stacks was used to demultiplex raw sequences by assigning individual reads to corresponding samples through unique ligated in‐line barcodes. After trimming verified barcodes (5–9 nucleotides), truncated 142 bp single‐end reads were checked for the presence of the *HindIII* restriction cut‐site sequence, and those with base call errors were discarded. Using a sliding window (15% of read length), reads showing an average decrease in quality score (Q score < 10; 90% base call accuracy) were also removed. Demultiplexed reads were then assembled into loci using stacks *denovo_map.pl *wrapper program with default parameters, with the exception of the following: *ustacks*: ‐m 2 ‐M 2 ‐‐bound_high 0.05; *cstacks*: ‐n 2. The parameters are defined as: m = minimum depth of coverage to create a stack, M = maximum distance (in nucleotides) allowed between stacks, and n = number of mismatches allowed between sample loci when building a catalog. Following completion of the assembly, a population map was used to assign individuals to their respective phenotypic group (Linne'vatn* *= Anadromous*, Normal, Dwarf; *Ellasjøen *=* *Littoral, Pelagic*), and stacks *populations *module was implemented, requiring loci to be present in ≥60% of individuals in each group with a minimum allele read depth of 10× (‐r 0.6 ‐m 10). To maintain independence of loci with multiple polymorphisms, only the first SNP within each locus was retained for downstream analyses (‐‐write_single_snp).

In an attempt to remove putative paralogous sequence variants (PSVs), the output vcf file (Danecek et al., 2011) from stacks *populations *module was used to identify PSVs using the method of McKinney, Waples, Seeb, and Seeb (2016). Each locus showing a proportion of heterozygotes >0.55 and/or an allele read depth ratio deviation ±5 were labeled as a probable PSV. A blacklist containing putative PSVs and SNPs with a global minor allele count (MAC) <3 was then supplied to stacks, and the *populations *module was run again using the same parameters as above. The filtered dataset was exported in plink format (Purcell et al., 2007), converted using pgdspider (v.2.0.5.2; Lischer & Excoffier, 2012), and hierfstat in R (v. 0.04‐22; Goudet, 2005) was used to calculate allelic richness using rarefied allele counts (*A*_R_), overall and per‐locus observed heterozygosity (*H*_OBS_), expected heterozygosity (*H*_EXP_), and inbreeding coefficients (*F*_IS_). Tests of heterozygote excess/deficit within each morph were conducted with default parameters using the exact test implemented in genepop (v.4.3; Rousset, 2008).

### 2.4 Identification of outlier loci

Delineation of both neutral genetic variation and adaptive genetic variation can be important for evaluating differentiation among populations (Funk, McKay, Hohenlohe, & Allendorf, 2012). While assessment of neutral variation can provide insight into stochastic processes as well as the demographic history of populations, evaluation of adaptive variation can help elucidate the role of selective processes in shaping population differentiation. We therefore sought to independently examine the contribution that neutral variation and adaptive variation have on phenotypic differentiation among Arctic charr morphs.

Using the full filtered dataset with individual phenotype supplied as prior information, neutral loci and those potentially under selection were identified with bayescan (v.2.1; Foll & Gaggiotti, 2008). Using default parameters and a prior of 100, the significance of candidate outlier loci was determined using a *q*‐value threshold of 0.05, which corresponds to a false discovery rate (FDR) of 0.05. To reduce type I error, results were verified with a coalescent‐based approach by identifying putative outliers exhibiting high *F*_ST_ values relative to neutrality as implemented in lositan v. 1.6 (Antao, Lopes, Lopes, Beja‐Pereira, & Luikart, 2008). The analysis was performed using 100,000 simulations, confidence intervals of 99%, and a FDR of 0.05. Since the accuracy of outlier detection programs is known to vary according to sample size and the observed pattern of genetic variation (Ahrens et al., 2018; Narum & Hess, 2011), outlier was required to be identified by both lositan and bayescan in order to be classified as putatively adaptive. These outlier loci are considred putaitvely adaptive until future validation studies are conducted (Shafer et al., 2015). A conservative approach was used for categorizing SNPs as neutral, using a p‐value range estimated by lositan of 0.10–0.90, and a *q‐*value threshold >0.50 as calculated by bayescan. A list containing putative adaptive and neutral SNPs were then used to extract each set of loci from the filtered dataset using plink v. 1.07.

### 2.5 Differentiation among morphs based on neutral loci

Pairwise linkage disequilibrium (LD) of neutral SNPs was estimated for each morph using plink. For each locus pair in strong LD (*r* ^2^ ≥ 0.80) in two or more morphs, the locus with the lowest genotyping frequency was discarded. The degree of pairwise genetic differentiation (*F*_ST_) was then examined between morphs at neutral loci. To determine whether pairwise comparisons significantly differed from zero, 1,000 bootstraps were used across all loci and 95% confidence intervals (95% CI) were generated using the diversity package in R (v. 1.9.9; Keenan, McGinnity, Cross, Crozier, & Prodöhl, 2013).

To further evaluate the genetic relationship among Arctic charr morphs, we used the successive *k*‐means clustering algorithm implemented in the R package adegenet (v.2.01; Jombart, 2008). After transforming the data using principal component analysis (PCA) to reduce the number of variables, *k*‐means clustering was used for increasing values of *K *(1–10), identifying the optimal number of clusters using the Bayesian information criterion (BIC). Using the inferred group assignment (based upon *k*‐means clustering), a discriminant analysis of principal components (DAPC) was then implemented, replacing missing values with overall mean allele frequencies and using the *optim.a.score* function to prevent over fitting the model (Jombart, 2008). The first two axes from the DAPC were then plotted using the R package ggplot2 (v. 3.0; Wickham, 2009). Results were also verified by estimating individual admixture proportions using the Bayesian clustering algorithm implemented in admixture (v.1.23; Alexander, Novembre, & Lange, 2009). Model complexity was estimated for each value of *k *(1–10) and evaluated using the cross‐validation function (*‐cv*) to identify the value of *k* with the lowest associated error.

### 2.6 Signatures of selection

In order to disentangle whether one or multiple morphs were driving any observed patterns of deviation from neutral expectation, three different approaches were used to attempt to resolve signatures of divergent selection. First, to provide a visual comparison of adaptive SNPs across all five morphs at each locus, allele frequencies were generated using genodive (v.2.0; Meirmans & Van Tienderen, 2004). Second, the degree of genetic differentiation (*F*_ST_) between morphs at adaptive SNPs was calculated using the same methods listed above (see differentiation among morphs section). Confidence intervals for neutral and adaptive pairwise *F*_ST_ values were then compared to infer evidence of divergent selection between morphs. Third, using the same methods listed above, bayescan was repeated using individuals from either Ellasjøen or Linne'vatn to test for repeated outliers at a local scale.

The 142 bp consensus sequence of each outlier was downloaded from the stacks catalog, converted to fasta format, and blastn (Johnson, Zaretskaya, RaytselisY, McGinnis, & Madden, 2008) was used to align and map the location of consensus outlier sequences with the *Salvelinus alpinus* reference genome (RefSeq: GCF_002910315.2). The position of the top alignment for each query sequence was examined for annotated regions within a 50 kb window using NCBI's *Salvelinus alpinus *genome browser (http://www.ncbi.nlm.nih.gov/genome/gdv/), and UniProtKB (http://www.uniprot.org/) was used to infer potential biological function.

## 3 RESULTS

### 3.1 SNP calling and quality filtering

Following library preparation and sequencing, 342,141,354 raw Illumina reads were evaluated using stacks *process_radtags* script. Of these reads, 321,417,116 (93.9% of total reads) were retained, with demultiplexed samples averaging 3.84 million reads (±0.91 *SD*). A preliminary stacks run identified two individuals with low genotyping rate (>80% missing data; 2 *Anadromous* samples), which were excluded from the analysis. Assembling loci across the remaining individuals (*N* = 89) resulted in retention of 12,271 loci, containing a total of 18,095 SNPs (mean = 1.5 SNPs/locus). After restricting the analysis to one SNP per locus, and excluding 49 SNPs that were homozygous, 1,308 SNPs were identified as putative paralogs (Supporting Information Figure S1). stacks *populations *module was then supplied a blacklist containing putative PSVs and SNPs with a global minor allele count (MAC) <3 (2,839 SNPs), resulting in a final matrix of 8,075 filtered SNPs for downstream analyses (Table 2).

**Table 2:** The number of loci remaining after filtering out paralogous sequence variants (PSVs) and SNPs with a minor allele count (MAC) <3. The final number of retained loci is shown for the full filtered dataset, and the neutral^a^ and adaptive datasets, as determined by agreement between bayescan and lositan

Filtering steps			Dataset		
SNPs	psvs	mac	Filtered	Neutral	Adaptive
12,271	10,914	8,075	8,075	5,976	17

a After removing four loci in linkage disequilibrium.

Overall, the filtered dataset had a mean coverage depth of 60× across all individuals and loci. When sample sizes were normalized by rarefaction for six diploid individuals, mean allelic richness (*A*_R_) was lower for Ellasjøen samples (*A*_R_ range = 1.373–1.384) compared to Linne'vatn samples (*A*_R_ range = 1.585–1.594), with little observable difference between morphs from the same geographic area (Table 1). Likewise, Ellasjøen samples had lower expected heterozygosity (*H*_E_ range = 0.153–0.159) compared to Linne'vatn samples (*H*_E_ range = 0.234–0.235; Table 1; Supporting Information Table S1). Inbreeding coefficients varied slightly among morphs from each lake (*F*_IS_ range = 0.021 to −0.041), with all morphs exhibiting significant evidence of heterozygote deficit (*p* = 0.00–0.001; Table 1).

### 3.2 Identification of outlier loci

Using the full filtered dataset (8,075 single SNP loci), bayescan identified 17 candidate outliers (0.21% of SNPs), with *q‐values *of candidate outliers ranging from 0.0002 to 0.049 (Table 2; Supporting Information Table S2). All of these 17 candidate outliers were also identified by lositan, which classified 418 SNPs as candidates for positive selection, with probabilities (simulated *F*_ST_ < sample *F*_ST_) ranging from 0.996 to 1.0. Overall, examination of bayescan and lositan output resulted in agreement of 17 putatively adaptive and 5,980 neutral SNPs (Table 2; Supporting Information Table S2), which were subsequently extracted from the filtered dataset using plink.

### 3.3 Differentiation among morphs based on neutral loci

Out of the 5,980 neutral SNPs, four SNPs were excluded based upon evidence of strong LD (*r* ^2^ > 0.8) in two or more morphs, with the remainder (5,976 SNPs) used to examine patterns of group differentiation and neutral population structure. Substantial variation in pairwise genetic differentiation was observed among morphs (*F*_ST_ range = 0.002–0.178). As expected, high differentiation was observed among allopatric morphs (*F*_ST_ = 0.152–0.178), with low to moderate differentiation among sympatric morphs (*F*_ST_ = 0.002–0.074; Table 3). All pairwise *F*_ST_ values were statistically significant from zero (based upon lower bound 95% CI), with the exception of the pairwise comparison between the sympatric *Littoral *and *Pelagic *morphs in Ellasjøen (*F*_ST_ = 0.002), which may be attributed to small sample sizes.

**Table 3:** Pairwise *F*_ST_ (95% confidence intervals) values using putatively adaptive (above diagonal, not shaded; 17 SNPs) and neutral loci (below diagonal, shaded gray; 5,976 SNPs^a^)

		Linne'vatn			Ellasjøen	
		*Anadromous*	*Normal*	*Dwarf*	*Littoral*	*Pelagic*
Linne'vatn	*Anadromous*		0.058 (0.007–0.108)*	0.511 (0.352–0.645)*	0.853 (0.771–0.924)*	0.834 (0.691–0.943)*
	*Normal*	0.020 (0.018–0.022)*		0.445 (0.305–0.569)*	0.851 (0.782–0.902)*	0.827 (0.697–0.922)*
	*Dwarf*	0.074 (0.070–0.077)*	0.058 (0.055–0.061)*		0.824 (0.720–0.908)*	0.808 (0.655–0.927)*
Ellasjøen	*Littoral*	0.178 (0.172–0.183)*	0.178 (0.172–0.184)*	0.174 (0.168–0.179)^**^		−0.040 (0.000–0.6336)
	*Pelagic*	0.154 (0.148–0.159)*	0.154 (0.148–0.159)*	0.152 (0.147–0.158)*	0.002 (−0.001–0.006)	

a After removing four loci in linkage disequilibrium.

* Significantly different from zero (based upon lower bound 95% CI).

**Figure 2:**
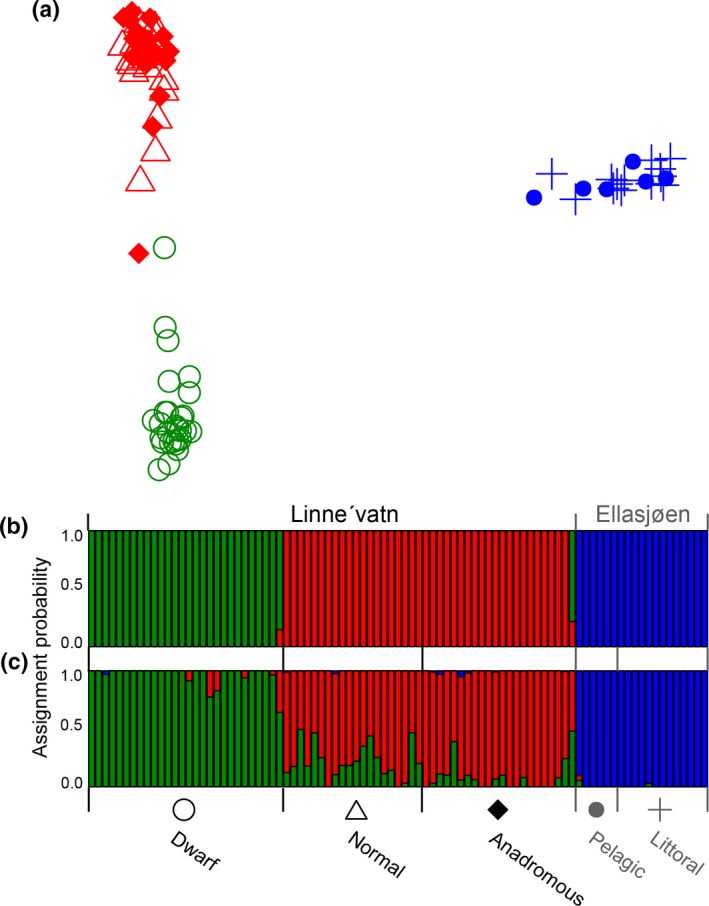
Cluster analyses of 5,076 neutral loci among five different Arctic charr morphs from lakes Ellasjøen and Linne'vatn: (a) first two axes from a discriminant analysis of principal components with points corresponding to individuals. Shape of points is based upon actual phenotypic group, with color corresponding to inferred group; (b) barplot of inferred group membership probability (*k = *3 clusters) from *k‐*means clustering; and (c) *k = *3 also identified by admixture as best describing the structure in the data

Evaluating the BIC scores following successive *k‐*means clustering identified *k = *3 as the optimal number of genetic groups in the dataset (Supporting Information Figure S2a). This inferred group size was used for the DAPC after retaining seven principal components (27.9% conserved variance), as identified using the *a‐score* optimization criterion. The first axis of the DAPC clearly discriminated between the allopatric morphs, whereas the second axis largely separated the *Dwarf* morph from the *Anadromous* and *Normal *morphs from Linne'vatn (Figure 2a). Based on cluster membership probabilities generated from the DAPC, samples from Ellasjøen grouped together (blue crosses and filled circles), whereas the *Anadromous *and *Normal *samples from Linne'vatn formed a distinct cluster (red diamonds and triangles), from the *Dwarf *samples (green empty circles; Figure 2b). However, one *Normal *and one *Anadromous* sample showed evidence of admixture with the *Dwarf* cluster, with membership probabilities of 0.15 and 0.78, respectively (Figure 2b). The cross‐validation function implemented in admixture also identified *k* = 3 as the most likely number of genetic clusters in the dataset (Supporting Information Figure S2). Individual admixture coefficients showed similar results to *k‐*means clustering, yet revealed additional overlap of the *Anadromous *and *Normal *morphs with the *Dwarf *morph (admixture range = 0.0001–0.641; Figure 2c).

### 3.4 Signatures of selection

Using individuals from both lakes, we identified 17 putatively adaptive SNPs supported by both bayescan and lositan. Similar to the set of neutral SNPs, high differentiation was observed among allopatric morphs (*F*_ST_ = 0.808–0.853), yet a substantial range in genetic differentiation occurred among sympatric morphs (*F*_ST_ = −0.04–0.511; Table 3). Again, all pairwise *F*_ST_ values were statistically significant from zero, with the exception of the comparison of the sympatric *Littoral *and *Pelagic* morphs in Ellasjøen (Table 3). Pairwise comparison of confidence intervals between adaptive and neutral SNPs illustrated that all allopatric morphs showed significant differences (i.e., confidence intervals did not overlap), while sympatric morphs only differed for the *Dwarf *versus *Anadromous *and *Dwarf *versus *Normal *comparisons (Supporting Information Figure S4).

Examining the distribution of allele frequencies for these 17 loci indicated that a majority (15/17) showed allele frequency disparities among allopatric morphs, largely driven by the presence of multiple monomorphic loci for Ellasjøen samples. Repeating bayescan using only the Ellasjøen samples failed to identify any outliers. Two loci (56596|Sequence 13 and 85782|Sequence 16) showed substantially different allele frequencies within the Linne'vatn samples (Supporting Information Figure S3), and repeating bayescan analysis using only the Linne'vatn samples identified these loci as repeat outliers at a local scale, with *q*‐values ranging from 0.0002 to 0.0003.

Using all of the consensus outlier sequences as queries (*n* = 17), blastn had solid hits against the *Salvelinus alpinus *reference genome, with E*‐*scores ranging from 9.00e‐21 to 6.00e‐67 (Supporting Information Table S3). Four outlier sequences aligned to an unplaced scaffold, two sequences aligned to a linkage group with no coding regions within a 50 kb window, and the remaining 11 sequences mapped to a genomic location containing one or more coding regions within a 50 kb window (Supporting Information Table S3). These targets for selection included genes associated with the regulation of transcription, regulation of telomeres, ion transport, iron binding, protein folding, embryonic development, bone morphogenesis, and memory (Supporting Information Table S3). The two local outliers (56596|Sequence 13 and 85782|Sequence16) identified within the Linne'vatn samples aligned to an unplaced scaffold and Linkage Group 6.1, respectively. The outlier sequence on Linkage Group 6.1 (85782|Sequence 16) mapped to within 50 kb of the interleukin 1 receptor accessory protein like 2 (IL1RAPL2), which is located on the X chromosome in humans and is linked to cognitive disability (Supporting Information Table S3). An important paralog of this gene is IL1RAPL1, which is expressed at a high level in the hippocampal memory system, suggesting a specialized role in the physiological processes underlying memory and learning abilities (www.genecards.org/cgi-bin/carddisp.pl?gene=IL1RAPL1).

## 4 DISCUSSION

### 4.1 Neutral and adaptive genomic differentiation

We found that landlocked Arctic charr in Ellasjøen had lower genetic diversity (i.e., mean allelic richness and expected heterozygosity) than those in Linne'vatn. Varying levels of genetic differentiation were detected among allopatric and sympatric morphs using 5,976 neutral and 17 adaptive loci. Fifteen of the identified adaptive loci were shared between Linne'vatn and Ellasjøen, and consensus sequences had solid hits against the *Salvelinus alpinus *reference genome (E*‐*scores range: 9.00e‐21–6.00e‐67; Supporting Information Table S3). Two adaptive loci were restricted to samples from Linne'vatn. Only one (85782|Sequence 16) of the two adaptive loci mapped to an annotated genomic location. We found one coding region within a 50 kb window of this locus, interleukin 1 receptor accessory protein like 2 (IL1RAPL2), which is located on the X chromosome in humans. While mutations in this gene have been shown to cause cognitive disabilities in humans, its role in fishes has yet to be determined. In contrast, we failed to detect *F*_ST_ outliers using only samples from Ellasjøen, suggesting that there were no sequenced adaptive genetic differences between the *Littoral *and *Pelagic* morphs. However, these findings should be considered preliminary given our limited sampling from Ellasjøen. A more comprehensive study focusing on all morphs within this system is definitely warranted.

### 4.2 Comparison of allopatric morphs

Pairwise *F*_ST_ estimates based on neutral and adaptive loci indicated high differentiation among allopatric morphs (Table 3), and cluster analyses of neutral loci separated samples from Linne'vatn and Ellasjøen (Figure 2). Considering that fish within Ellasjøen are landlocked, the neutral and adaptive genetic differences observed between morphs from the two lakes were expected. High genetic differentiation based on neutral markers has also been previously reported for six landlocked populations of Arctic charr across Fennoscandia (Shikano et al., 2015), and 12 landlocked populations in Maine (Bernatchez et al., 2002). Like Linne'vatn and Ellasjøen (Bøyum & Kjensmo, 1978; Klemetsen et al., 1985), these lakes are of postglacial origin (<12,000 years old) but it is uncertain if any have been geographically isolated for a similar length of evolutionary time as Ellasjøen (Bernatchez et al., 2002; Shikano et al., 2015). Our findings suggest that the allopatric morphs in Linne'vatn and Ellasjøen are locally adapted, and thus, responses to climate change will be variable between the lakes.

### 4.3 Comparison of sympatric morphs

We could not genetically distinguish the sympatric *Littoral *and *Pelagic *morphs in Ellasjøen, indicating that they represent a single breeding population. The *F*_ST_ values based on both neutral and adaptive loci were statistically insignificant (Table 3), and cluster analysis of neutral loci failed to distinguish any subgroups of genetic variation within Ellasjøen (Figure 2). Interestingly, previous research differentiated between the *Littoral *and *Pelagic* morphs based on foraging differences as well as different sensitivities to photoperiod variation (Hawley et al., 2016, 2017). Our genetic results from Ellasjøen, however, are consistent with other genetic studies which found that certain sympatric Arctic charr morphs are not genetically or reproductively distinct (see review Jonsson & Jonsson, 2001; e.g., Nordeng, 1983). However, the failure to detect genetic differentiation (i.e., neutral or adaptive) between the *Littoral *and *Pelagic* morphs in Ellasjøen may reflect inadequate sampling of these sympatric morphs. Future genetic investigations of Ellasjøen charr should also include the previously described dwarf morph (Hawley et al., 2016) to determine whether those individuals represent a genetically distinct group. A more comprehensive investigation, sampling additional individuals and all morphs in Ellasjøen, should indicate whether adaptive differences are present among these morphs. If our preliminary genetic results are accurate though, Arctic charr in Lake Ellasjøen may be more resilient to climate change than expected. For instance, a single population exhibiting substantial variation in morphology, foraging preferences, and photosensitivity (Hawley et al., 2016, 2017) may adapt more readily to increased light penetration and elevated freshwater productivity (Heino et al., 2009; Michelutti et al., 2005; Prowse et al., 2006; Woodward et al., 2010) than a set of smaller, fragmented populations with distinct phenotypes.

Within Linne'vatn, cluster analysis of neutral loci failed to distinguish the *Anadromous *and resident *Normal* morphs (Figure 2). The *F*_ST_ values estimated for neutral and adaptive loci were both moderate and statistically significant between these sympatric morphs, although differences were somewhat higher for adaptive loci (Table 3). This result is not necessarily a surprise, as anadromous and resident Arctic charr both overwinter in freshwater, and the relationship between migratory behavior and gene flow is known to vary (Moore et al., 2013). To assess the opportunity for reproduction between morphs, otolith chemistry, tagging, and radio telemetry (albeit logistically challenging in the Arctic environment) could be used to determine the spatial and temporal patterns in migration and spawning of the *Anadromous *and resident *Normal *morphs in Linne'vatn (e.g., Brenkman & Corbett, 2007).

Our genetic results for the *Anadromous *and resident *Normal *morphs in Linne'vatn are consistent with previous studies of Arctic charr in the Salangen River, Norway, which suggested that anadromous and resident individuals belonged to the same population (Nordeng, 1961, 1983). In addition, Nordeng (1983) conducted extensive rearing experiments and found that a fraction of the resident Arctic charr always transformed into anadromous fish. That said, a higher proportion of the offspring from anadromous parents smoltified (i.e., underwent physiological changes to adapt to seawater) when released into the river compared to offspring from resident parents, indicating that there is some genetic influence on anadromy (Nordeng, 1983).

Climate change is predicted to increase light penetration, water temperature, and carbon dioxide concentrations for freshwater systems (Heino et al., 2009; Michelutti et al., 2005; Prowse et al., 2006; Woodward et al., 2010). Such changes are expected to enhance freshwater productivity, making the risk of predation while feeding at sea more costly. As a result, the frequency of resident Arctic charr may increase (Finstad & Hein, 2012; Jensen et al., 2018; Reist, Wrona, Prowse, Dempson et al., 2006). Our GBS approach (Elshire et al., 2011) only sequenced a fraction of the Arctic charr genome, but if results are representative, a reduced number of *Anadromous *individuals relative to resident *Normal *morph may not result in a significant loss of genetic diversity. However, anadromous Arctic charr represent a valuable fishery compared to freshwater residents, particularly for indigenous Arctic communities (Christiansen & Reist, 2013; Moore et al., 2013; Tallman et al., 2018). Therefore, there are considerable socioeconomic concerns if anadromous individuals across the Arctic become less frequent due to climate change.

In contrast to our findings for *Anadromous* and *Normal* Arctic charr, we found strong evidence for reduced gene flow and adaptive differences between the non‐migratory *Dwarf *morph and the *Anadromous *and resident *Normal* fish in Linne'vatn. *F*_ST_ values were moderate for neutral sites but high for putatively adaptive loci (Table 3). Cluster analysis of neutral loci distinguished *Dwarf *Arctic charr from the *Anadromous *and *Normal *morphs, although some shared genotypes may indicate genetic drift or a degree of introgression among all three morphs within Linne'vatn (Figure 2). Our findings are consistent with other studies of sympatric variation in Arctic charr that also found evidence for strong genetic differentiation between *Dwarf *individuals and larger sized morphs (e.g., Adams et al., 2008; Alekseyev et al., 2014; Gordeeva et al., 2015; Samusenok et al., 2006).

Previous studies have documented differences in breeding time between typical‐sized and dwarf morphs from other lakes (Jonsson & Hindar, 1982; Pavlov & Osinov, 2008). A similar “adaptation by time” (Hendry & Day, 2005) could explain the genetic differentiation observed for the *Dwarf *morph in Linne'vatn. It is possible that assortative mating based on spawning site or depth could also be an important driver of reproductive isolation among sympatric morphs (Jonsson & Hindar, 1982). Alternatively, like many profundal morphs of Arctic charr from other lakes, the *Dwarf *charr in Linne'vatn may be an example of paedomorphosis where juvenile traits are retained during adulthood (Danzmann et al., 1991; Jonsson & Jonsson, 2001; Klemetsen et al., 2003; Knudsen et al., 2006; Parsons et al., 2011; Skoglund et al., 2015; Skùlason et al., 1989). If *Dwarf *individuals are paedomorphs, it may be physiologically or behaviorally difficult for matings to occur with *Anadromous *or *Normal *individuals, substantially limiting the opportunity for gene flow.

The high *F*_ST_ values for adaptive loci indicate that there may be substantial divergence between the *Dwarf *morph and *Anadromous *and resident *Normal *individuals. Aside from developmental differences due to the possibility of paedomorphosis, traits such as foraging preferences and habitat usage are likely to be influential. In other lakes, foraging differences can be subtle, but dwarf Arctic charr are often more generalist feeders (Adams et al., 2008; Samusenok et al., 2006) or focused upon benthos (Alekseyev et al., 2014; Fraser et al., 1998). Larger morphs often cannibalize dwarf or small‐sized morphs (Knudsen et al., 2016; Samusenok et al., 2006). Svenning et al. (2007) found that the diet of small individuals in Linne'vatn (<15 cm, predominantly *Dwarf*) was strongly characterized by chironomid *Oliveridia tricornis *(Oliver, 1976) larvae, whereas larger Arctic charr (>15 cm; *Anadromous *and *Normal*) within the lake were mainly cannibals (Svenning et al., 2007). These feeding differences could affect habitat use and the potential for gene flow, given that cannibalism by *Anadromous *and *Normal *morphs is potentially mortal risk to the reproductive success of *Dwarf *individuals. If cannibalism during spawning is common, we would expect selection to be strong against *Dwarf *individuals mating outside of their morph, unless such matings provide increased fitness, which could lead to the evolution of sexual cannibalism (Elgar & Schneider, 2004). In future studies, it would be useful to examine the rates of breeding and cannibalism between larger sized and *Dwarf* Arctic charr to determine whether this is a plausible factor to have affected gene flow.

Overall, the *Dwarf *morph represents a genetically distinct component of Arctic charr diversity in the Linne'vatn. Considering similar genetic evidence from other studies of *Dwarf *morphs (Alekseyev et al., 2014; Gordeeva et al., 2015; Samusenok et al., 2006), the potential response of small or profundal resident morphs to climate change warrants further investigation. It should be noted, however, that elevated freshwater temperatures are predicted to result in a lengthened growing season for Arctic charr (Budy & Luecke, 2014). If *Dwarf *individuals are not restricted to small body sizes due paedomorphosis or other heritable genetic changes, it is possible that *Dwarf *individuals could reach large enough sizes to compete or reproduce more often with larger morphs (Budy & Luecke, 2014). Such a change could lead to increased introgression among morphs and a reduction in overall genetic diversity in Linne'vatn. Further research is needed to determine whether *Dwarf* individuals are restricted to small body sizes, and whether increased freshwater temperature affects the growth rate of the morph.

## 5 CONCLUSIONS

The results of this study demonstrate the value of utilizing putatively adaptive loci to investigate intraspecific variation. Geographically isolated, allopatric populations of Arctic charr are likely to be genetically distinct, but genetic divergence among sympatric morphs varies, and response of populations and morphs to climate change probably needs to be considered on a case‐by‐case basis. That said, results from *Anadromous *and *Normal *morphs in Linne'vatn indicate that migratory differences do not necessarily indicate substantial genetic differences within a single lake. In concordance with previous studies (Alekseyev et al., 2014; Gordeeva et al., 2015; Samusenok et al., 2006), *Dwarf *Arctic charr in Linne'vatn were the most genetically distinct group observed, and the response of *Dwarf *morphs to climate change therefore warrants further investigation. Given that we observed substantial adaptive and neutral genetic diversity between just two lakes in the geographically remote Svalbard Archipelago, out results suggest that Arctic charr exhibit significant genetic diversity across the Arctic. The impact of continued climate change upon the diversity of other Arctic anadromous and freshwater fish species, and in turn, the wider ecosystem, fisheries, and affected indigenous communities, requires further investigation.

## AUTHOR CONTRIBUTIONS

K.G.O. and A.N.B. designed the experiments; K.G.O. and A.N.B. performed the experiments; K.G.O. and A.N.B. analyzed the data; and K.G.O., A.N.B., and F.V. wrote the manuscript.

## Data Availability

Three genepop files representing the filtered dataset (8,075 loci), the neutral dataset (5,976 loci), the adaptive dataset (17 loci), and a script depicting all code used in stacks pipeline, analyses, and figure generation. Dryad DOI: https://doi.org/10.5061/dryad.8hq6qv2.
