# Supplementary figures and images for: Characterizing neutral and adaptive genomic differentiation in a changing climate: The most northerly freshwater fish as a model
Source: Ecol Evol. 2019 Jan 15;9(4):2004–17. doi: 10.1002/ece3.4891 (PMC6392408; doi:10.1002/ece3.4891)

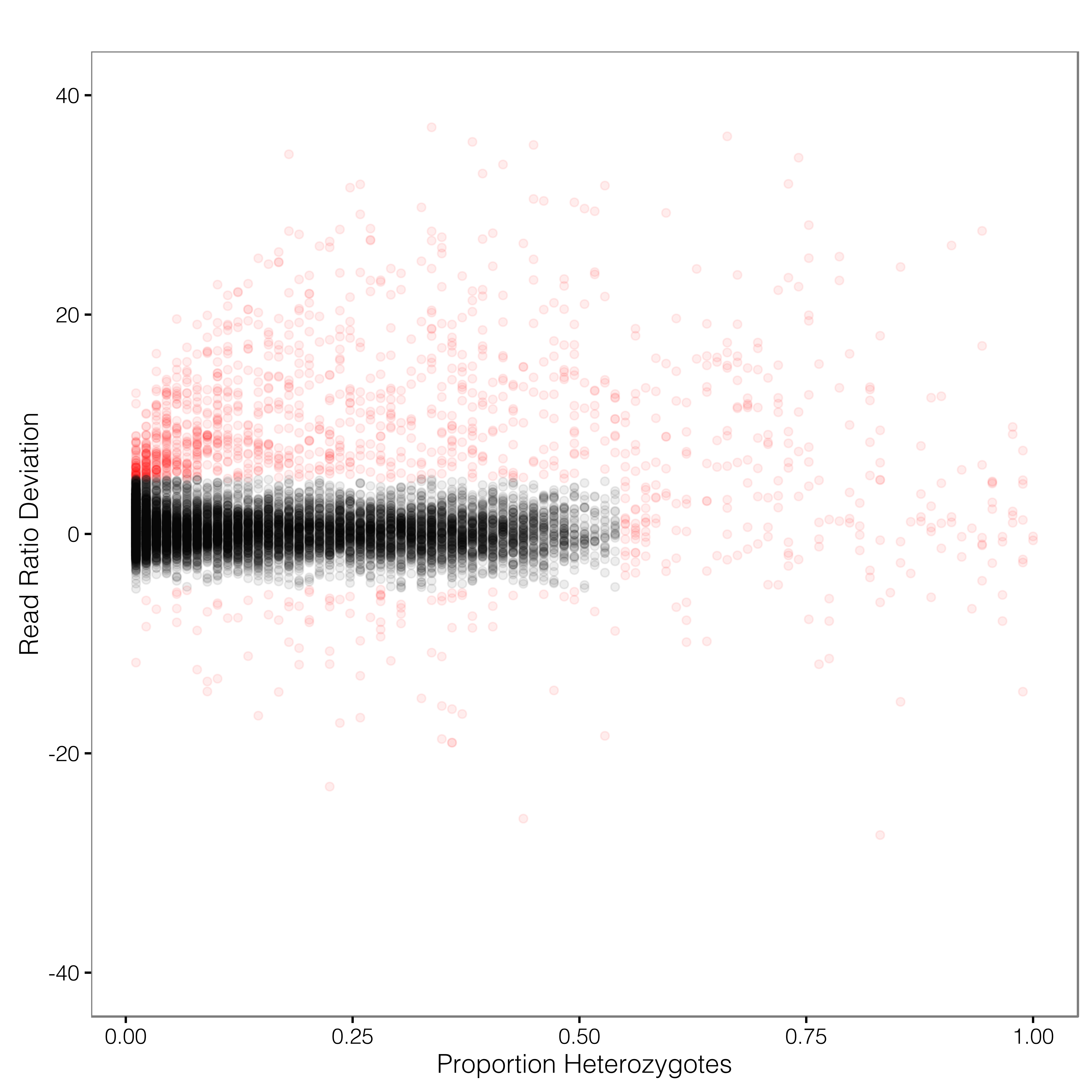

Supplement: Supplementary file 1 [file ECE3-9-2004-s001.png]

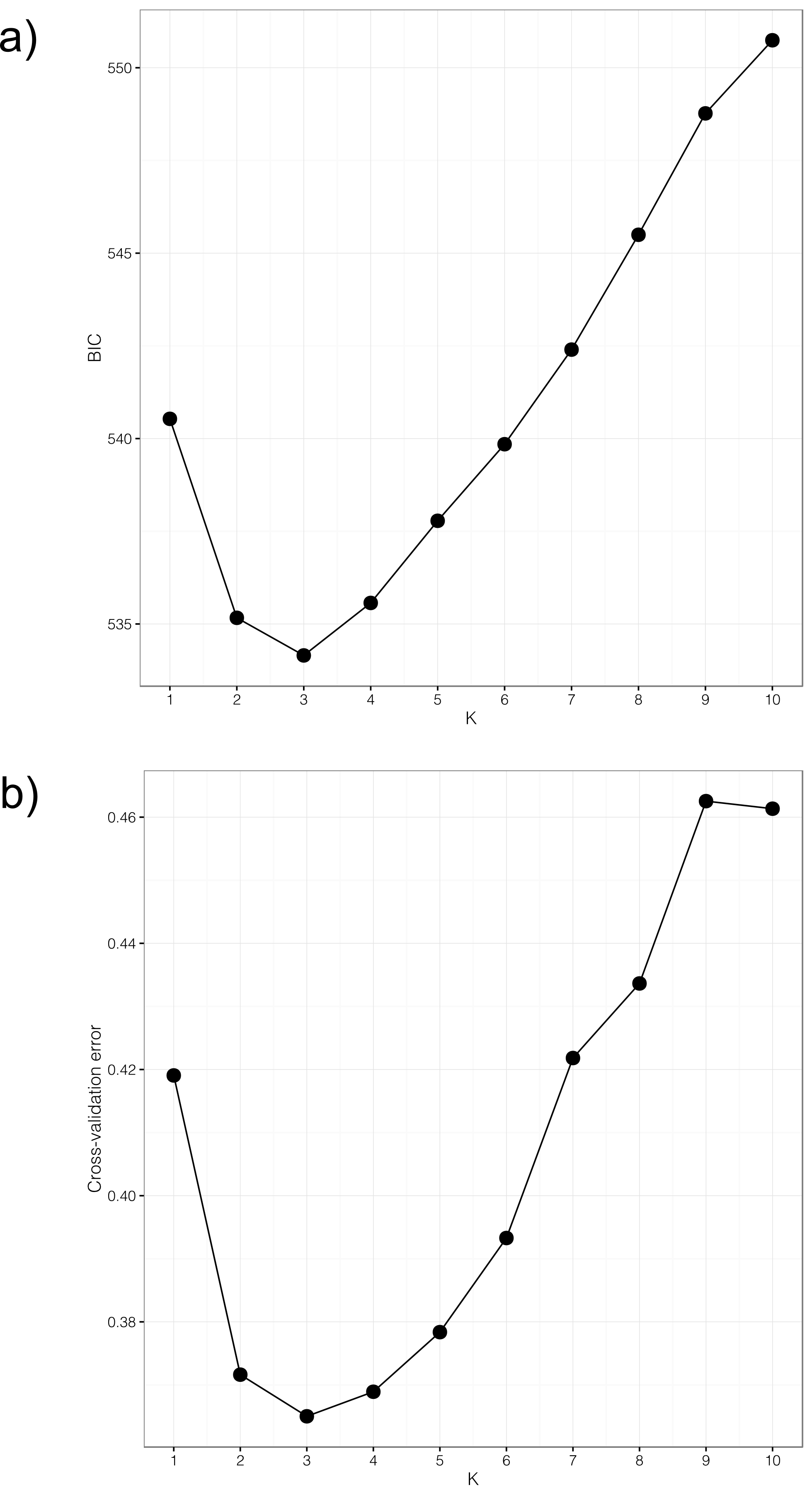

Supplement: Supplementary file 2 [file ECE3-9-2004-s002.png]

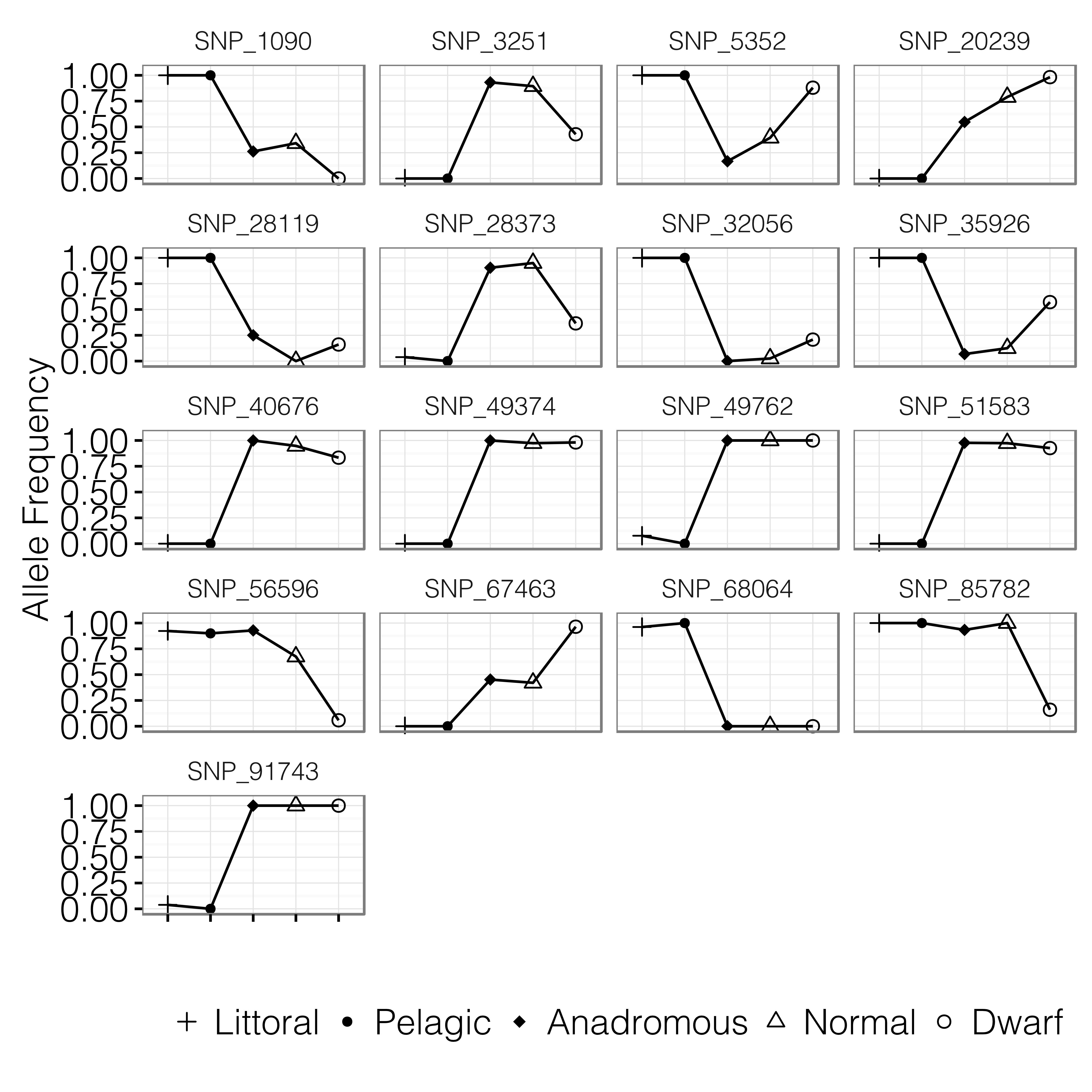

Supplement: Supplementary file 3 [file ECE3-9-2004-s003.png]

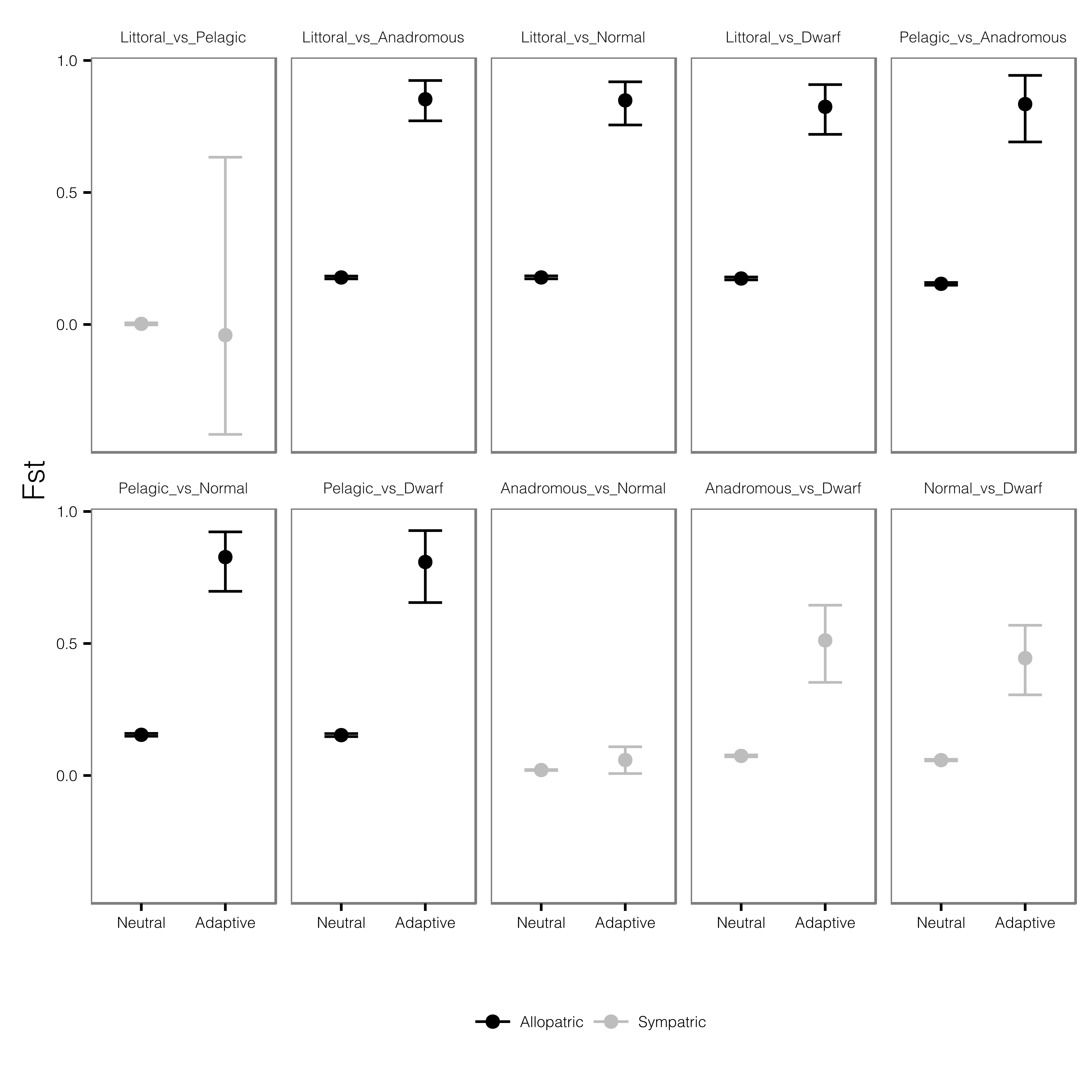

Supplement: Supplementary file 4 [file ECE3-9-2004-s004.png]
